# Laboratory and Greenhouse Evaluation of a Granular Formulation of *Beauveria bassiana* for Control of Western Flower Thrips, *Frankliniella occidentalis*

**DOI:** 10.3390/insects10020058

**Published:** 2019-02-20

**Authors:** Xingrui Zhang, Zhongren Lei, Stuart R. Reitz, Shengyong Wu, Yulin Gao

**Affiliations:** 1 State Key Laboratory for Biology of Plant Diseases and Insect Pests, Institute of Plant Protection, Chinese Academy of Agricultural Sciences, Beijing 100193, China; xy20126043@163.com (X.Z.); zrlei@ippcaas.cn (Z.L.); 2 Department of Crop and Soil Sciences, Malheur County Extension, Oregon State University, Ontario, OR 97914, USA; Stuart.Reitz@oregonstate.edu

**Keywords:** Western flower thrips, Fungal granules, Scanning electron microscopy, Fluorescence microscopy, Soil-treatment, Biological control

## Abstract

Western flower thrips (WFT) is one of the most important pests of horticultural crops worldwide because it can damage many different crops and transmit various plant viruses. Given these significant impacts on plant production, novel methodologies are required to maximize regulation of WFT to minimize crop losses. One particular approach is to develop control strategies for the non-feeding, soil-dwelling stages of WFT. Control of these stages could be enhanced through the use of granules impregnated with entomopathogenic fungi mixed in the soil. The use of soil-applied fungi contrasts with existing approaches in which entomopathogenic fungi are formulated as oil-based suspensions or water-based wettable powders for foliar applications against the feeding stages of WFT. To examine the efficacy of this approach, we evaluated the effects of a granular formulation of *Beauveria bassiana* on the soil-dwelling, pupal phases of *Frankliniella occidentalis* in laboratory bioassays and greenhouse experiments. Based on micromorphological observations of fungal conidia during the infection process after treatment of WFT with a *B. bassiana* suspension, fungal conidia complete the process of surface attachment, germination, and penetration of the body wall of the WFT pupa and enter the host within 60 h of treatment. Given these results, we undertook a controlled greenhouse experiment and applied *B. bassiana* granules to soil used to cultivate eggplants. Populations of *F. occidentalis* on eggplants grown in treated soil were 70% lower than those on plants grown in the untreated soil after 8 weeks. Furthermore, when measuring the survival and growth of *B. bassiana* on granules under different soil moisture conditions, survival was greatest when the soil moisture content was kept at 20%. These results indicate that the application of *B. bassiana*-impregnated granules could prove to be an effective biological control strategy for use against *F. occidentalis* under greenhouse conditions.

## 1. Introduction

Western flower thrips (WFT), *Frankliniella occidentalis* (Pergande) (Thysanoptera: Thripidae), is one of the most important pests of horticultural crops worldwide [1,2,3]. Damage is caused directly through oviposition (aesthetic damage to fruiting crops) and feeding (damage scars to foliage, flowers and fruits) [4]. Moreover, WFT serves as an important vector of several species of damaging plant viruses in the genus *Tospovirus* (Bunyaviridae) [5], including *tomato spotted wilt virus* (TSWV) and *impatiens necrotic spot virus* (INSV), which may cause over 90% yield loss in some instances [6,7,8].

The life cycle of WFT contains the foliar-feeding (adult, first and second larval instars) and soil-dwelling developmental stages (late second larval instars, pre-pupae, and pupae). Ninety-eight percent of the WFT that pupate enter the soil although this is dependent on the host plant species [9,10]. However, the majority of current pest management strategies focus on control of adults and larvae with a substantial portion of the soil-dwelling stages being neglected [11,12]. Consequently, the use of chemical insecticides remains commonplace for managing WFT, with particularly intensive use and application occurring in high-value crops such as pepper, tomato and eggplant [13]. However, frequent applications of chemicals may leave excessive pesticide residues on the plant, result in high levels of insecticide resistance, and are particularly expensive [3,14,15,16,17].

As an alternative option for control, entomopathogenic fungi are well-documented biological control agents and have great potential for controlling agricultural insect pests worldwide [18]. Successful applications include the use of *Beauveria bassiana* (Balsamo) Vuillemin (Hypocreales: Cordycipitaceae), *Metarhizium brunneum* (Metchnikoff) Sorokin, *Isaria fumosorosea* (Wize) A.H.S. Br. and G. Sm and *Lecanicillium lecanii* R. Zare and W. Gams targeting Lepidoptera, Coleoptera, Hemiptera, and Diptera [19]. For example, *B. bassiana* has been shown to be a highly effective biological control agent against the different life stages of WFT under laboratory, greenhouse and open-field conditions [20,21,22].

Most formulations of entomopathogenic fungi have been developed for foliar applications to control the foliar feeding stages of WFT [12,16,23,24,25]. The efficacy of fungal sprays is likely to be low against the soil-dwelling stages of the pest which are protected from direct exposure to sprays of fungal conidia. However, bioassays using soil-incorporated, air-dried microsclerotia of *Metarhizium anisopliae* preparations resulted in significant infection and mortality in larvae of the sugar beet root maggot, *Tetanops myopaeformis* [26]. Applications of millet-based *B. bassiana* granules on rice nursery soil can be an effective and efficient biological control strategy for the management of rice water weevils [27]. The application of *B. bassiana* granules to the soil could provide enhanced control of the below-ground stages of WFT through better targeting of infective conidia [11]. 

This current research was designed to test the feasibility of using granular formulations of *B. bassiana* strain GZGY-1-3, a native fungal isolate previously shown to be effective against the foliar stages of WFT, to control the pest in the soil. A series of steps were undertaken: (1) laboratory bioassays of the granules to confirm the efficacy of this approach and to define a suitable field application rate; (2) observations of fungal attachment and infection using scanning electron microscopy and fluorescence microscopy to confirm the susceptibility of WFT pupae to this fungal strain and determine the dynamics of the infection process; and (3) an evaluation of fungal granules to control WFT in greenhouse eggplants. We wished to test the overarching hypothesis that the application of *B. bassiana* granules to the soil would provide effective control of WFT under laboratory and greenhouse conditions.

## 2. Materials and Methods

### 2.1. Rearing Protocols for WFT

A colony of WFT was established with adults and collected from pepper fields (*Capsicum annuum* L.) in the Mentougou Vegetable Planting Center of Beijing, China, in 2013 (39°56′25 N, 116°06′05 E). Unsterilized kidney bean pods (*Phaseolus vulgaris* L. (Fabaceae)) contained within tube-shaped glass jars (0.5 L) with Snap-On lids were used to culture the WFT colonies. A 10-cm diameter hole was cut in each lid and covered with a fine mesh to allow for ventilation. These tubes were maintained under controlled conditions of 25 ± 1 °C, 70 ± 10% RH and a L14:D10 h photoperiod. A method of feeding by age was utilized to maximize colony growth. To do this, bean pods were replaced every three days because WFT take approximately 3 days to complete each larval stage at 25 °C. To prepare an even-aged cohort of WFT pre-pupae for the lab trials, eight bean pods carrying WFT eggs were removed from the colony. Newly emerged first instar WFT were removed and allowed to develop in synchrony for three days and were then transferred to a new jar containing fresh bean pods. After approximately seven days, WFT pre-pupae were collected in the lower part of the rearing device and were used in the experiments that are described below.

### 2.2. Fungal Strains and Culture

*Beauveria bassiana* isolate, GZGY-1-3 (maintained at the China General Microbiological Culture Collection Center No. 9254; GenBank Accession Number KP994951), was used in the study. This strain was originally isolated from an infected cadaver of *Ostrinia furnacalis* in Guizhou, China. The fungus was cultured on Sabouraud Dextrose Agar (SDA) (HuanKai Microbial, Guangzhou, Guangdong, China) at 26 ± 1 °C for seven days and harvested from the plates in sterile aqueous 0.05% Tween 80 (Medchemexpress, South Brunswick, NJ, USA). A suspension containing 1 × 10^7^ conidia/ml was prepared from the stock suspension, which was used in the microscopic studies (to inoculate the pre-pupae) and to inoculate the solid medium when producing granules. The viability of the conidia was confirmed as being over 90%, using protocols described by Goettel and Inglis [28]. Previous studies have shown that this isolate is virulent to WFT at this concentration [29].

### 2.3. Production of Beauveria bassiana Granules

To obtain granules for experimentation, *B. bassiana* was grown through a liquid–solid two-phase fermentation process. During the liquid phase, *B. bassiana* conidia obtained from agar slant culture media were used to inoculate a spore-forming medium (2% agar powder, 2% wheat bran, 0.5% peptone, 0.1% NH_4_NO_3_, 0.3% KH_2_PO_4_, 0.1% MgSO_4_·7H_2_O) for strain activation at 26 °C for 5 d until conidiation occurred. Conidia were harvested from the medium, and a suspension containing 1 × 10^7^ conidia/ml was prepared using the methods outlined above, which was used for the microscopic studies (to inoculate the pre-pupae). Three hundred milliliter liquid growing medium (35 g glucose, 15 g yeast powder, 0.037 g/L KCl, 0.25 g/L MgSO_4_·H_2_O, 1.20 g/L NaH_2_PO_4_, 1 L distilled water) was dispensed to each 1 L Erlenmeyer flask (48 in total). Each flask was inoculated with the 1 × 10^7^ conidia/ml conidial suspension and incubated on a rotary shaker at 150 rpm 26 °C for 3 d. Mycelia and blastospores were produced in the liquid medium. The solid substrate (wheat bran and rice husk at a ratio of 4:1, 0.3% KH_2_PO_4_, 0.1% MgSO4·7H2O, 0.1% NH_4_NO_3_) was sterilized by autoclaving at 121 °C for 30 min. To inoculate the solid substrate, 1.5 L blastospore culture containing 1 × 10^11^ conidia/ml was used, and this was then transferred to the bag (12 in total). Approximately 3 kg of solid substrate was placed in a fermentation gunny bag (Chengxin Textile Co., Ltd., Changge, Henan, China) with proper ventilation (74 cm × 107 cm) and maintained under controlled conditions of 26 °C for 10 d. A 500 watt electric fan (Midea Group, Foshan, Guangdong, China) continuously worked in the first 3 days to ensure that the substrate received ‘proper ventilation’ in the gunny bags. For the first three days, ambient humidity was maintained at over 90% to stimulate mycelium growth. Thereafter, the humidification process was stopped to promote conidiation. After 10 days, the solid medium was placed in a YC-6 commercial ebullated dryer (Yongchang Granulating Drying Equipment Co., Ltd., Changzhou, Guangdong, China), and conidia were separated from the substrate. The substrate was mixed with vermiculite at a 3:1 ratio to produce granules for the experiments. The viability of the conidia retained on the granules was over 90%, which resulted in a conidial loading of 1 × 10^8^ per gram of granules.

### 2.4. Pathogenicity Assay of Beauveria bassiana Granules

The efficacy of the *B. bassiana* granules was investigated against WFT pre-pupae under laboratory conditions. Loam soil was collected from a greenhouse facility at the Langfang Experiment Station of the Chinese Academy of Agricultural Sciences, Langfang, China and dried using an electric constant temperature blast drying oven (Jinghong, Shanhai, China) at 105 °C for 8 h. *B. bassiana* granules (1 × 10^8^ conidia per gram) were mixed with soil at six rates: 0, 6.25, 12.5, 25, 50 and 100 g per kg, and 100 g of each soil-granule mixture was placed in each of the 3 Petri dishes for each soil-granule mix (12-cm diameter). Then, 20 pre-pupae were transferred into each Petri dish and a fresh kidney bean pod was added as food. Dishes were covered with a plastic film that was perforated to provide ventilation. Petri dishes were kept in the chamber at 25 ± 1 °C, RH 70 ± 5% and a L14:D10 h photoperiod. The number of adult WFT was counted after 5 days, and the experiment was repeated three times.

### 2.5. Micromorphological Observations of Fungal Infection

To investigate the infection process of WFT pre-pupae by *B. bassiana,* 500 pre-pupae were infected by dipping in a conidial suspension (1 × 10^7^ conidia/ml, 0.05% aqueous Tween 80) for 5 s. This concentration is commonly used for spray applications of the fungus against WFT in greenhouses in China. After treatment, 20 pre-pupae were transferred to each of the 6 Petri dishes (7-cm diameter) containing bean leaf discs (7-cm diameter) on moist filter paper; the dishes were covered with plastic film that was perforated to provide ventilation. WFT were then removed from the dishes at prescribed time points, i.e., 2 h, 24 h, 36 h, etc. The Petri dishes were maintained in an environmental chamber at 25 ± 1° C, RH 70 ± 5%, and a L14:D10 photoperiod.

To prepare infected WFT pre-pupae for examination by scanning electron microscopy (Olympus China Corporation, Beijing, China), 20 WFT exposed to *B. bassiana* were removed from the Petri dishes 2, 24, 36, 48, 60, and 72 h after inoculation and placed in 5% glutaraldehyde in cacodylate buffer for 12 h at 25 °C. The samples were treated according the method of Wu et al. (2016) [30] and examined under high vacuum mode on a Quanta 200 FEG SEM at magnifications of 500× to 20,000×. 

Similarly, inoculated WFT pre-pupae were examined by fluorescence microscopy. A fluorescent dye stock solution was prepared by dissolving 4 mg FDA (Sigma-Aldrich, St. Louis, MO, USA) in 1 mL of acetone; 35 mL of FDA stock solution was then diluted in 4 mL of deionized water to prepare a fluorescent dye working solution. The working solution for each experiment was freshly prepared and the vessel containing the working liquid was shielded from light by aluminum foil and kept on ice.

Twenty *Beauveria bassiana*-treated pre-pupae were removed from each Petri dish 2, 12, 24, 36, 48, and 72 h after inoculation, and were placed on a clean glass slide. A single drop of working solution was placed on the insect which was overlaid with a coverslip. Fifteen treated insects of each group were observed by excitation light of 450–490 nm under a blue filter fluorescent microscope (Olympus China Corporation, Beijing, China) at 400× magnification.

### 2.6. Colonization of Soil by Beauveria bassiana under Different Soil Moisture Conditions

The soil moisture content (defined herein as the percentage of water calculated by weight contained in 100 g dry soil) was adjusted to 10%, 15%, 20%, 25%, and 30%, respectively. Ten grams of *B. bassiana* granules was mixed with 100 g of dried loam soil and water was added to achieve the desired moisture content. Each batch was transferred to a Petri dish which was covered with plastic film. Three replicate batches (dishes) were prepared per treatment. The dishes were transferred to square plastic boxes containing a small amount of water in the bottom to maintain soil moisture levels through the trial and then kept at 25 °C. Soil samples were collected from the dishes at random every day for 8 weeks using a 1-cm diameter core sampler. To assess the level of soil colonization by *B. bassiana*, the number of colony-forming units (CFU) per gram of dried soil was determined. Three grams of each soil sample was weighed into a 250 mL flask which contained 97 mL of sterile aqueous 0.05% Tween 80. The contents were mixed thoroughly on a rotary shaker at 195 RPM/min for 2 h. A dilution series was prepared from the resulting suspension, and 300 μL samples from each dilution were plated onto PDA medium supplemented with streptomycin; three replicate plates were prepared from each dilution. Plates were incubated at 25 °C for 4 days. Plates with between 10 and 100 colonies were selected, and colonies were counted and used for data analysis [31]. Concurrently, a second 3 g soil sample was dried in a constant temperature blast drying oven at 105 °C for 8 h. The sample was then re-weighed, providing the relative dry weight of the soil, allowing the CFUs to be calculated and expressed per gram of dried soil.

### 2.7. Greenhouse Experiments

Experiments examining the effectiveness of the granular formulation of *B. bassiana* against *F. occidentalis* were conducted using eggplants (*Solanum melongena* L. (Solanaceae)) in a greenhouse facility at the Langfang Experiment Station of the Chinese Academy of Agricultural Sciences, Langfang, China (32°22′48 N, 34°55′58 E). Briefly, eggplant seedlings were transplanted at the three-leaf stage in a total of 13 double-rows, 14 plants per row. Two weeks after transplanting, groups of four adjacent eggplants were covered with cages constructed with fine-mesh thrips-proof screening (length × width × height = 70 cm × 110 cm × 2 m). The cages were fitted with a zipper to allow the plants to be accessed and enable *F. occidentalis* populations to be monitored. At the beginning of the experiment, 200 adult WFT were added to each cage to establish a breeding population. One hundred and fifty grams of *B. bassiana* granules per cage were applied to the soil surface and were incorporated into the top 1 cm of soil within the row using a hoe. This concentration of *B. bassiana* granules was shown to cause 80% mortality of WFT in the laboratory bioassays described earlier. The eggplant was watered by drip irrigation. The soil moisture content was measured by a soil humidity sensor (Dihui Technology, Beijing, China), and the experimental soil was maintained at 20% to simulate the optimal conditions for *B. bassiana* activity. This level of soil moisture was also suitable for eggplant growth. The granular treatment and an untreated control were replicated three times within the greenhouse. 

A five-point sampling method was used to count the number of WFT. Specifically, leaves were selected by visually dividing the eggplants in a cage into three equal vertical strata. One leaf was randomly sampled from each stratum of each plant, giving a total of 12 leaf samples from each cage and all WFT on the leaves (upper and lower leaf surfaces) were counted in situ. This ensured uniform sampling among plants and an accurate representation of the WFT population on each plant [30]. The initial population of WFT was counted before the start of the experiment and then counted each week thereafter for seven successive weeks.

### 2.8. Data Analysis

For the screening tests, mortality data were corrected with Abbott’s formula [32], normalized using arcsine square-root transformation and then subjected to analysis of variance (ANOVA) with means separated by the Tukey’s mean separation test at *p* = 0.05 to determine significance. For colonization of *B. bassiana* tests, a two-way repeated-measures ANOVA (‘proc GLM’ procedure in SAS 9.4) (first factor: the soil moisture content (five levels), second factor: sampling date (eight levels)) was used to compare the CFUs of *B. bassiana* between treatments over time. For the greenhouse trials, a two-way repeated-measures ANOVA (‘proc GLM’ procedure in SAS 9.4) (first factor: Fungus (application, untreated control), second factor: Sampling date (seven levels)) was used to compare the density of *F. occidentalis* between treatments over time. Differences in mean *F. occidentalis* densities between treatments were compared by t-test on each sampling date. All data were analyzed using SAS 9.4 at a 0.05 level of significance.

## 3. Results

### 3.1. Pathogenicity of Beauveria bassiana Granules to Frankliniella occidentalis

**Figure 1:**
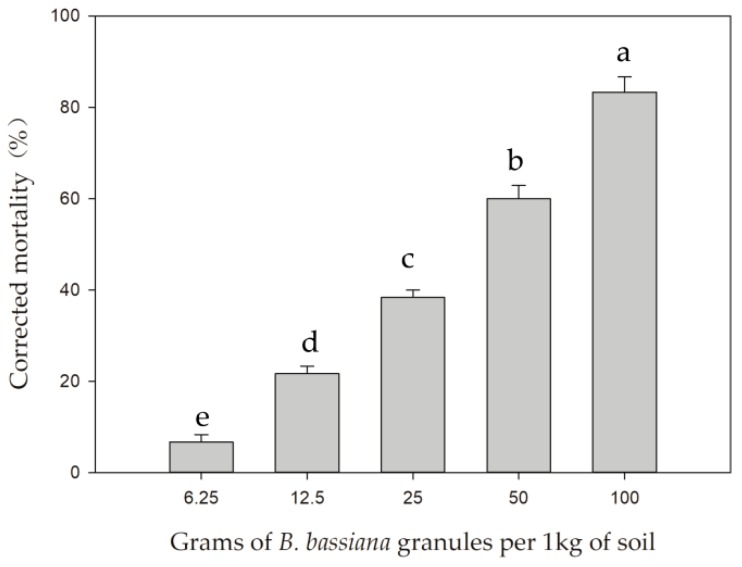
Effect of different concentrations of *Beauveria bassiana* granules on the mortality of soil-dwelling pupae of Western Flower Thrips, *Frankliniella occidentalis*. Columns marked with different letters are significantly different from one another. Bars above columns denote standard errors of the means.

Compared with the control, *B. bassiana* granules exerted significantly greater control efficacy against WFT. Mortality of *F. occidentalis* increased with increasing concentrations of *B. bassiana* (Figure 1). The highest level of WFT mortality was obtained from the treatment where 100 g of *B. bassiana*-containing granules was mixed with 1 kg of soil (83.33 ± 3.33%). There were significant differences between the five groups of treatments (F = 156.61; df = 4; *p* < 0.0001). Mycelia of *B. bassiana* were observed on the surface of WFT cadavers when dead thrips were examined under a stereomicroscope 4 days after infestation. These results confirmed that *B. bassiana* granules could be applied to the soil to control the soil-dwelling stages of WFT.

### 3.2. Micromorphological Observations of Fungal Infection

**Figure 2:**
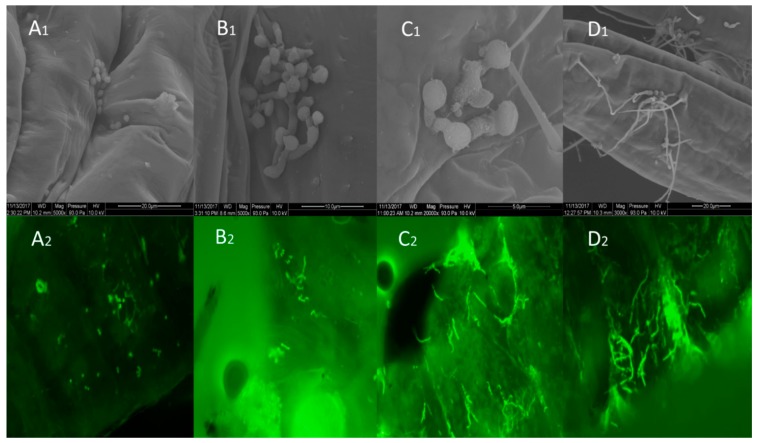
*Beauveria bassiana* GZGY-1-3 infecting pre-pupae of *Frankliniella occidentalis* as shown by scanning electron microscopy (SEM) (A1–D1) and fluorescence microscopy (A2–D2). A1 and A2: Conidia attached to the cuticle of *F. occidentalis* 2 h after immersion. B1 and B2: Germination of conidia on the insect cuticle. C1 and C2: Development of appressoria by *B.* *bassiana* germ tubes after 36 h. D1 and D2: Growth of *B. bassiana* hyphae over the surface of the pre-pupae after 60 h.

Scanning electron microscopy (SEM) and fluorescence microscopy revealed that conidia of strain GZGY-1-3 became attached to the body surface of WFT pre-pupae within 2 h (Figure 2A1,A2). After 24 h, conidia had germinated, and the germ tube extended over the insect cuticle (Figure 2B1,B2). The germ tubes formed infection structures (appressoria) and penetration of the cuticle occurred (Figure 2C1,C2), and a large number of conidia germinated and produced hyphae (Figure 2D1,D2). Mycelia that were observed covering the body within 60 h originated from the conidia on the surface of the pre-pupae. Mycelia were observed growing out of the insects within 72 h. These results indicated that GZGY-1-3 had the capacity to adhere to the WFT body and was pathogenic to it.

### 3.3. Colonization of Beauveria bassiana under Different Soil Water Concentrations

**Figure 3:**
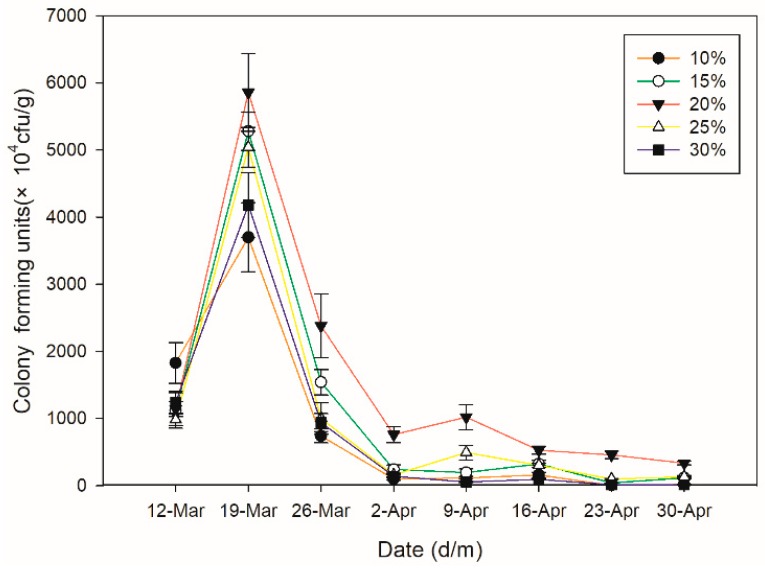
Effects of the soil moisture content on the survival and multiplication of *Beauveria bassiana.* Bars denote standard errors of the means. The first sample date shown is one week after inoculation of the soil with *B. bassiana*-impregnated granules.

The number of *B. bassiana* CFUs recovered from inoculated soil varied among the different soil moisture levels tested. The highest CFU levels (5.86 × 10^7^ CFU/g) were obtained in the 20% soil moisture treatments by week 2 (Figure 3), representing an increase of 414% over levels in week 1. Thereafter, the number of CFUs slowly declined. However, at week 5, the number of CFUs increased slightly and was 55% higher than levels in week 1, and greater than all other treatments, i.e., other soil moisture levels. At the 10% soil moisture content, the number of CFUs increased by 102% by week 2 and was lower than levels in all other soil moisture treatments. By week 8, CFUs had fallen to approximately 1 × 10^4^ CFU/g, and levels were comparable to those in the 30% soil moisture content treatments. There were significant differences in fungal levels among the different soil water treatments (F = 22.39; df = 4; *p* < 0.0001), and with time after treatment (F = 296.90; df = 7; *p* < 0.0001). Interestingly, there was also a significant interaction between the soil moisture content and treatment time (F = 2.95; df = 28; *p* < 0.0001).

### 3.4. Efficacy of Soil-Applied Beauveria bassiana Granules against Frankliniella occidentalis in a Greenhouse

**Figure 4:**
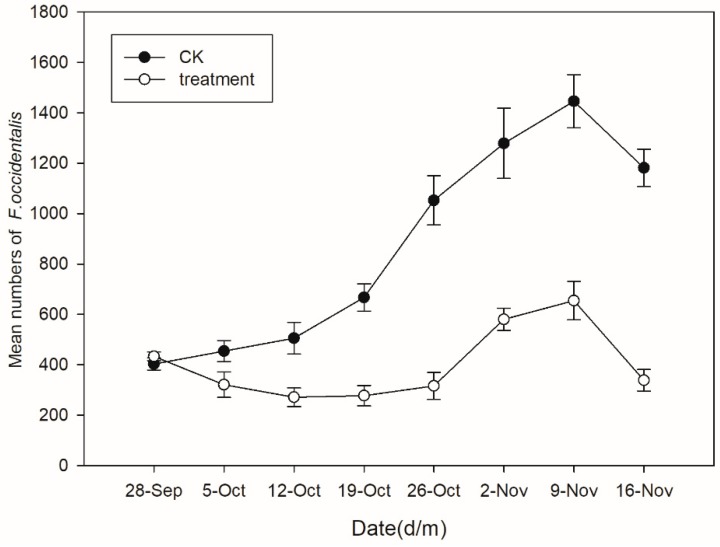
Mean number of *Frankliniella occidentalis* on greenhouse-grown eggplant soil treated with *Beauveria bassiana* granules or left untreated.

In the greenhouse experiments, soil moisture levels were maintained at 20% throughout to simulate optimal conditions for *B. bassiana* survival and persistence and WFT populations were significantly lower on plants grown in soil treated with the GZGY-1-3 granules than in the untreated control at all time points after plants were infested (Figure 4). There was no difference in the initial WFT populations across all treatments (t = −2.03; df = −2.03; *p* = 0.1117). After the application of *B. bassiana* granules, populations in the treatment group decreased significantly (t = −7.49; df = 4; *p* = 0.0017) within the first week. The granules continued to suppress WFT for eight weeks relative to the control. Both the treatment and control groups showed an overall upward trend in WFT numbers after four weeks, and WFT populations were highest after seven weeks (untreated control: 1445.67 ± 105.18 WFT/cage; treatment: 654.33 ± 76.50 WFT/cage). After eight weeks, WFT numbers declined by 67% in the *B. bassiana* treatment and there was a significant difference between the two populations (t = −7.49; df = 4; *p* = 0.0017). There were significant differences between treatments and times and there was a significant interaction of *B. bassiana* between time after treatment (Treatment: F = 43.99; df = 1; *p* = 0.0027; Time: F = 58.70; df = 7; *p* < 0.0001; Treat × Time: F = 24.60; df = 7; *p* < 0.0001).

## 4. Discussion 

In this study, applications of *B. bassiana* granules to the soil surface suppressed *F. occidentalis* population growth. Moreover, this research clarified the time taken for *B. bassiana* to infect the pupal stages of WFT and the influence of soil moisture conditions on *B. bassiana* survival and soil colonization. Our results confirm earlier reports that *B. bassiana* granules are an effective formulation of this entomopathogen for *F. occidentalis* control in soil and validated the potential of this alternative management approach [11].

Scanning and fluorescence electron microscopy were instrumental in observing the interactions between *B. bassiana* and *F. occidentalis*. We observed that the surface of the insect body becomes covered with hyphae, suggesting that *B. bassiana* strain GZGY-1-3 was pathogenic to WFT [33,34]. A large number of fungal conidia were adhered to the WFT cuticle in samples processed 2 h after immersion in the *B. bassiana* suspension, confirming the utility of fluorescence microscopy as a viable means of observing the fungal infection process in WFT [35]. We observed appressoria formation at the end of the germ tube using scanning electron microscopy. Appressoria are well-known infection structures involved in the penetration of the insect cuticle. Our observations agree with those of Wu et al. (2014) who demonstrated that most fungal germlings produce appressoria 24–48 h after contacting the cuticle of a susceptible insect [34]. Penetration pegs then develop from the appressoria and the insect body wall is breached through a combination of mechanical pressure and enzymatic degradation of the cuticular layers; toxins are then produced leading to the death of the infected insect [36,37].

*Beauveria bassiana* infection is moderated by external environmental factors, such as ambient humidity and temperature, which has limited use of *B. bassiana* in large-scale field settings. When environmental conditions are unsuitable, the conidia are easily inactivated, reducing their capacity to regulate pest populations [38]. Importantly, when fungal conidia are sprayed on plant leaves, they may be inactivated by sunlight and ultraviolet light or simply die due to rapid drying [11,39]. In contrast, when conidia are applied to the soil, they may be better protected from sunlight and high ambient temperatures, and relative humidity can be maintained to improve the survival and germination of the fungus [11]. 

Moisture is an important factor affecting spore germination, mycelial growth and the pathogenicity of *B. bassiana* [40,41]. It has been reported that efficacy increased with soil moisture but declined once soils became saturated for *B. bassiana* [41,42]. Mortality of WFT increased with increasing concentrations of *B. bassiana* in our trial to determine whether the *B. bassiana* granules were capable of infecting and controlling WFT. We found that the survival of *B. bassiana* conidia and the growth of the fungus from granules were significantly reduced when the soil moisture content was below 20%. 

In our greenhouse trials, granular treatments suppressed WFT population growth. Infection of WFT pre-pupae probably occurred as the insects moved into the soil. The formation of conidia on WFT cadavers could serve as a source of fresh inoculum capable of infecting susceptible insects in the same environment. The granular formulation clearly provided adequate protection against WFT in our experimental system and may be useful in other greenhouse vegetable production systems. Although a single biological control agent may not be expected to control an entire target pest population, IPM methods that combine multiple agents are likely to succeed [16]. Our research supports the use of *B. bassiana* as part of an integrated management approach. For instance, entomopathogenic fungal granules could be applied to the soil to eliminate the soil-dwelling stages of WFT, allowing insecticides to be applied for the additional management of WFT populations on the foliage when required [12,24]. Moreover, fungal granules could be combined with other natural enemies of WFT, including predatory mites, rove beetles and minute pirate bugs to improve the control effects [43].

## 5. Conclusions

In conclusion, our research demonstrates that the application of *B. bassiana* granules to the soil surface can successfully suppress WFT under greenhouse conditions. More in-depth research to devise viable application strategies for fungal granules and their integration with other biocontrol strategies could lead to a valuable alternative strategy for managing WFT. 

## References

[1] Reitz S.R. (2009). Biology and ecology of the western flower thrips (Thysanoptera: Thripidae): The making of a pest. Fla. Entomol..

[2] Mouden S., Sarmiento K.F., Klinkhamer P.G., Leiss K.A. (2017). Integrated pest management in western flower thrips: Past, present and future. Pest Manag. Sci..

[3] Gao Y.L., Lei Z.R., Reitz S.R. (2012). Western flower thrips resistance to insecticides: Detection, mechanisms and management strategies. Pest Manag. Sci..

[4] Cloyd R.A. (2016). Western flower thrips (Thysanoptera: Thripidae) and insecticide resistance: An overview and strategies to mitigate insecticide resistance development. J. Entomol. Sci..

[5] Moritz G., Kumm S., Mound L. (2004). Tospovirus transmission depends on thrips ontogeny. Virus Res..

[6] Robb K.L. (1989). Analysis of *Frankliniella occidentalis* (Pergande) as a Pest of Floricultural Crops in California Greenhouse. Ph.D. Dissertation.

[7] Pappu H.R., Jones R.A.C., Jain R.K. (2009). Global status of Tospovirus epidemics in diverse cropping systems: Successes achieved and challenges ahead. Virus Res..

[8] Webster C.G., Reitz S.R., Perry K.L., Adkins S. (2011). A natural M RNA reassortant arising from two species of plant-and insect-infecting Bunyaviruses and comparison of its sequence and biological properties to parental species. Virology.

[9] Steiner M.Y., Spohr L.J., Goodwin S. (2011). Relative humidity controls pupation success and dropping behaviour of western flower thrips, *Frankliniella occidentalis* (Pergande) (Thysanoptera: Thripidae). Aust. J. Entomol..

[10] Holmes N., Bennison J., Maulden K., Kirk W. (2012). The pupation behaviour of the western flower thrips, *Frankliniella occidentalis* (Pergande). Acta Phytopathol. Entomol. Hung..

[11] Skinner M., Gouli S., Frank C.E., Parker B.L., Kim J.S. (2012). Management of *Frankliniella occidentalis* (Thysanoptera: Thripidae) with granular formulations of entomopathogenic fungi. Biol. Control..

[12] Wraight S.P., Ugine T.A., Ramos M.E., Sanderson J.P. (2016). Efficacy of spray applications of entomopathogenic fungi against western flower thrips infesting greenhouse impatiens under variable moisture conditions. Biol. Control.

[13] Nault B.A., Shelton A.M. (2010). Impact of insecticide efficacy on developing action thresholds for pest management: A case study of onion thrips (Thysanoptera: Thripidae) on onion. J. Econ. Entomol..

[14] Zhao X.Y., Reitz S.R., Yuan H.G., Lei Z.R., Paini D.R., Gao Y.L. (2017). Pesticide-mediated interspecific competition between local and invasive thrips pests. Sci. Rep..

[15] Zhang T., Reitz S.R., Wang H.H., Lei Z.R. (2015). Sublethal effects of *Beauveria bassiana* (Ascomycota: Hypocreales) on life table parameters of *Frankliniella occidentalis* (Thysanoptera: Thripidae). J. Econ. Entomol..

[16] Reitz S.R., Yearby E.L., Funderburk J.E., Stavisky J., Momol M.T., Olson S.M. (2003). Integrated management tactics for *Frankliniella* thrips (Thysanoptera: Thripidae) in field-grown pepper. J. Econ. Entomol..

[17] Wu S.Y., Zhang Z.K., Gao Y.L., Xu X.N., Lei Z.R. (2016). Interactions between foliage- and soil-dwelling predatory mites and consequences for biological control of *Frankliniella occidentalis*. Biocontrol.

[18] Shah P.A., Pell J.K. (2003). Entomopathogenic fungi as biological control agents. Appl. Microbiol. Biotechnol..

[19] de Faria M.R., Wright S.P. (2007). Mycoinsecticides and mycoacaricides: A comprehensive list with worldwide coverage and international classification of formulation types. Biol. Control.

[20] Ludwig S.W., Oetting R.D. (2002). Efficacy of *Beauveria bassiana* plus insect attractants for enhanced control of *Frankliniella occidentalis* (Thysanoptera: Thripidae). Fla. Entomol..

[21] Jacobson R., Chandler D., Fenlon J., Russell K. (2001). Compatibility of *Beauveria bassiana* (Balsamo) Vuillemin with *Amblyseius cucumeris* Oudemans (Acarina: Phytoseiidae) to control *Frankliniella occidentalis* Pergande (Thysanoptera: Thripidae) on cucumber plants. Biocontrol Sci. Technol..

[22] Ansari M., Brownbridge M., Shah F., Butt T. (2008). Efficacy of entomopathogenic fungi against soil-dwelling life stages of western flower thrips, *Frankliniella occidentalis*, in plant-growing media. Entomol. Exp. Appl..

[23] Wu S.Y., Zhen H., Wang E.D., Xu X.N., Lei Z.R. (2017). Application of *Beauveria bassiana* and *Neoseiulus barkeri* for improved control of *Frankliniella occidentalis* in greenhouse cucumber. Crop Prot..

[24] Wu S.Y., Gao Y.L., Xu X.N., Zhang Y.P., Wang J., Lei Z.R., Smagghe G. (2013). Laboratory and greenhouse evaluation of a new entomopathogenic strain of *Beauveria bassianafor* control of the onion thrips *Thrips tabaci*. Biocontrol Sci. Technol..

[25] Ugine T.A., Wraight S.P., Sanderson J.P. (2006). Influences of impatiens pollen and exposure to *Beauveria bassiana* on bionomics of western flower thrips *Frankliniella occidentalis*. Biol. Control.

[26] Jackson M.A., Jaronski S.T. (2009). Production of microsclerotia of the fungal entomopathogen *Metarhizium anisopliae* and their potential for use as a biocontrol agent for soil-inhabiting insects. Mycol. Res..

[27] Kim J.S., Lee S.J., Skinner M., Parker B.L. (2014). A novel approach: *Beauveria bassiana* granules applied to nursery soil for management of rice water weevils in paddy fields. Pest Manag. Sci..

[28] Goettel M.S., Inglis G.D. (1997). Fungi: Hyphomycetes. Manual of Techniques in Insect Pathology.

[29] Li Y.P., Lei Z.R., Wang H.H. (2013). Selection of *Beauveria bassiana* strains against *Frankliniella occidentalis* and their conidial production characteristics. Chin. J. Biol. Control.

[30] Wu S.Y., Gao Y.L., Smagghe G., Xu X.N., Lei Z.R. (2016). Interactions between the entomopathogenic fungus *Beauveria bassiana* and the predatory mite *Neoseiulus barkeri* and biological control of their shared prey/host *Frankliniella occidentalis*. Biol. Control.

[31] Li Y.L., Wang Y.Y., Dong J.Z., Zhou T., Li J. (2012). The effects on survival ability of *Beauveria bassiana* under soil microecological environment. Chin. Agric. Sci. Bull..

[32] Abbott W.S. (1925). A method of computing the effectiveness of an insecticide. J. Econ. Entomol..

[33] Wu S.Y., Gao Y.L., Zhang Y.P., Wang E.D., Xu X.N., Lei Z.R. (2014). An entomopathogenic strain of *Beauveria bassiana* against *Frankliniella occidentalis* with no detrimental effect on the predatory mite *Neoseiulus barkeri*: Evidence from laboratory bioassay and scanning electron microscopic observation. PLoS ONE.

[34] Holder D.J., Keyhani N.O. (2005). Adhesion of the entomopathogenic fungus *Beauveria* (Cordyceps) *bassiana* to substrata. Appl. Environ. Microbiol..

[35] Wang D.J., Lei Z.R., Wang S.Y., Wang H.H. (2015). A new fluorescent microscopy method for identifying *Beauveria bassiana* infected *Bemisia tabaci* nymphs. Chin. J. Appl. Entomol..

[36] Charnley A., Leger R.S. (1991). The role of cuticle-degrading enzymes in fungal pathogenesis in insects. The Fungal Spore and Disease Initiation in Plants and Animals.

[37] Gillespie J.P., Bailey A.M., Cobb B., Vilcinskas A. (2000). Fungi as elicitors of insect immune responses. Arch. Insect Biochem..

[38] Fargues J., Luz C. (2000). Effects of fluctuating moisture and temperature regimes on the infection potential of *Beauveria bassiana* for *Rhodnius prolixus*. J. Invertebr. Pathol..

[39] Rangel D.E.N., Anderson A.J., Roberts D.W. (2008). Evaluating physical and nutritional stress during mycelial growth as inducers of tolerance to heat and UV-B radiation in *Metarhizium anisopliae* conidia. Mycoll. Res..

[40] Hallsworth J.E., Magan N. (1999). Water and temperature relations of growth of the entomogenous fungi *Beauveria bassiana*, *Metarhizium anisopliae*, and *Paecilomyces farinosus*. J. Invertebr. Pathol..

[41] Fuxa J.R., Richter A.R. (2004). Effects of soil moisture and composition and fungal isolate on prevalence of *Beauveria bassiana* in laboratory colonies of the red imported fire ant (Hymenoptera: Formicidae). Environ. Entomol..

[42] Shipp J.L., Zhang Y., Hunt D.W.A., Ferguson G. (2003). Influence of humidity and greenhouse microclimate on the efficacy of *Beauveria bassiana* (Balsamo) for control of greenhouse arthropod pests. Environ. Entomol..

[43] Saito T., Brownbridge M. (2016). Compatibility of soil-dwelling predators and microbial agents and their efficacy in controlling soil-dwelling stages of western flower thrips *Frankliniella occidentalis*. Biol. Control.

